# Supplementary material for: A High-Quality De novo Genome Assembly from a Single Mosquito Using PacBio Sequencing
Source: Genes (Basel). 2019 Jan 18;10(1):62. doi: 10.3390/genes10010062 (PMC6357164; doi:10.3390/genes10010062)
Supplement: Supplementary file 1 [file genes-10-00062-s001.pdf]

Supplementary Material

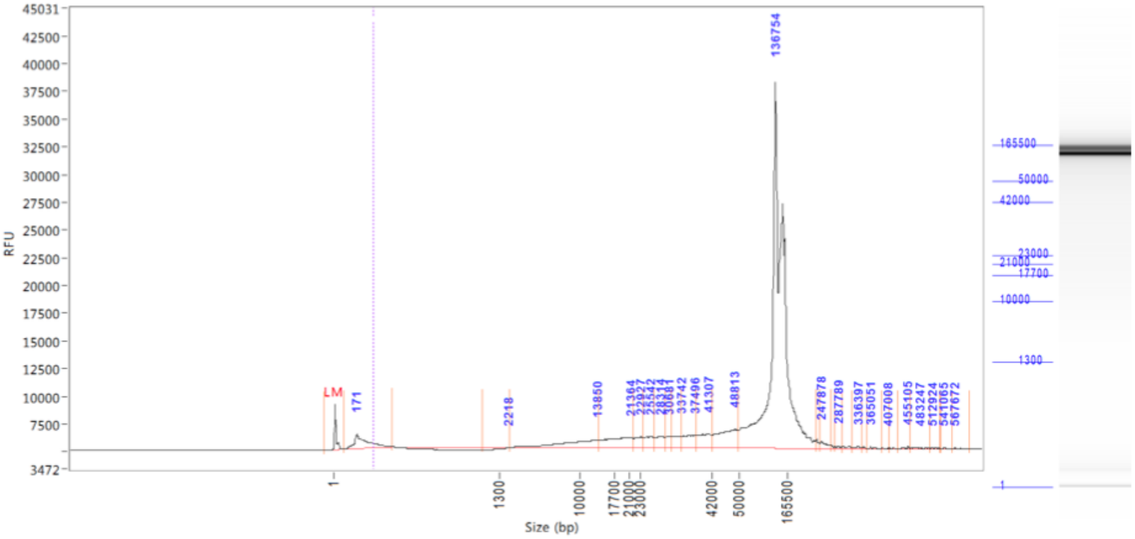

Figure S1. Femto Pulse evaluation of the Modified MagAttract DNA extraction prior to shipment to California.

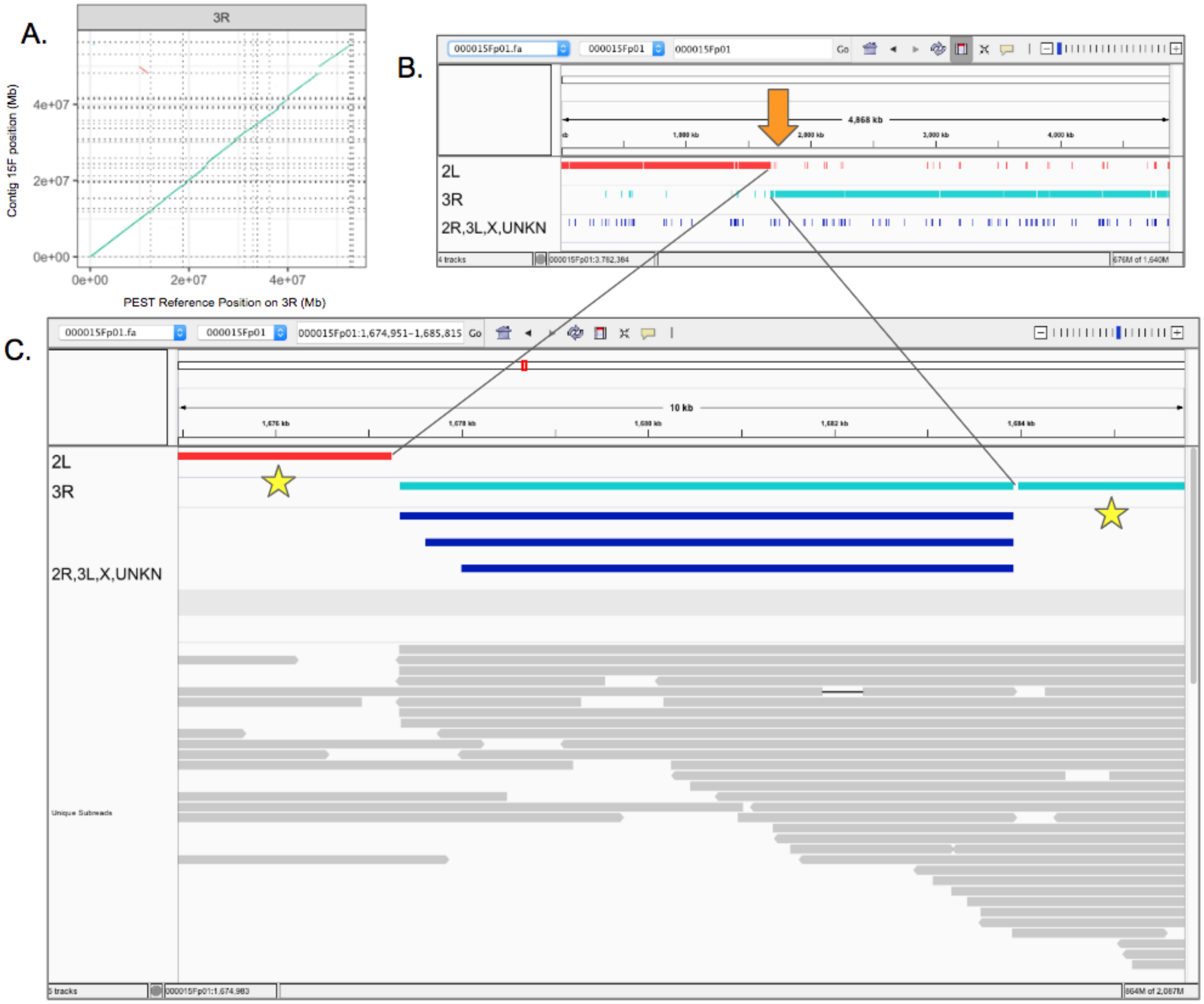

Figure S2. A chimeric contig between 2L and 3R. A. Alignment of PacBio contigs to PEST identifies a candidate chromosomal rearrangement. B. IGV screenshot of breakpoint (orange arrow) localized by alignment of contig to PEST. Red: alignment to 2L, turquoise: alignments to 3R, navy blue: alignments to other chromosomes and unplaced contigs. C. IGV visualization of mapped unique subreads at breakpoint shows 0 subreads mapping across the central repetitive region into the unique flanking sequence on the left (2L) and right (3R) (stars). A count of spanning reads was also determined with bedtools bamtobed utility. The 6.5kb central region aligns to four loci in the PEST genome and has ~370 bp of sequence similarity to the Tc1-like transposase gene in *An. gambiae*.

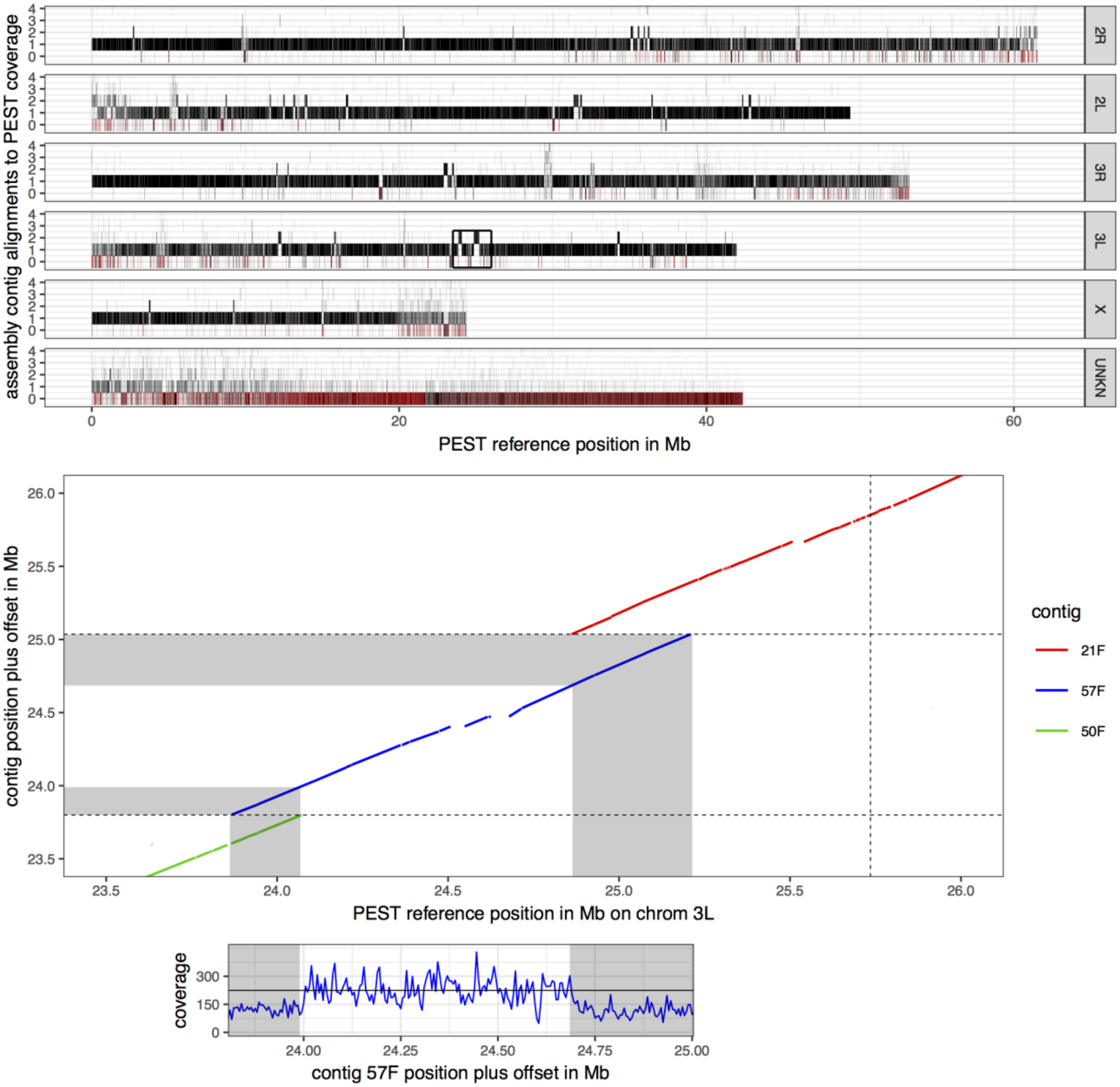

Figure S3. Alignment and coverage plot (top) of the PacBio assembly contigs relative to PEST, and magnification of one area of excess coverage (bottom). In the top panel, the number of alignments of PacBio contigs to PEST are represented by black bars, with most of the genome showing a 1:1 correspondence to PEST. Red denotes N's in the reference. Isolated areas of higher number of contig alignments are visible, one of which (black box) is magnified in the bottom panel. Here, the ends of neighboring contigs overlap, which is currently not resolved with the Purge Haplotigs software since the overlap is only partial. The sequencing depth of PacBio reads for the central (blue) contig (57F) corroborate this interpretation, exhibiting half of the expected coverage in the greyed regions of contig overlap, and with the corresponding ends of the red and green contigs complementing with the other half of coverage, respectively (not shown for clarity).

	1 Cell	2 Cells	3 Cells
**Total bases (Gb)**	23.6	48.5	72.7
**Total unique bases (Gb)**	4.46	8.31	12.8
**Unique coverage**	17 X	31 X	45 X
**Assembly size (Mb)**	150	265	271
**Number of contigs**	3,290	815	580
**Contig N50 (Mb)**	0.066	1.5	3.5

Table S1. Statistics for *Anopheles coluzzii de novo* genome assemblies as a function of the number of SMRT Cells used for the assembly.

Loading concentration	Gb/ Cell	Mean Polymerase Read Length	N50 Polymerase Read Length	Mean Subread Length	N50 Subread Length	P0	P1	P2
5 pM	24.1	40290	116615	8185	12978	26.0%	60.1%	13.9%
5 pM	23.6	40077	114807	8254	13132	27.1%	59.0%	14.0%
6 pM	25.0	47177	122898	8012	12751	35.3%	53.1%	11.7%

Table S2. Run statistics for the three Sequel SMRT Cells.

Gene Count (%)	Raw PacBio	Curated PacBio	Previous Reference
Complete	2745 (98.0)	2747 (98.1)	2448 (87.5)
Complete Single Copy	2635 (94.1)	2680 (95.7)	2446 (87.4)
Complete Duplicated	110 (3.9)	67 (2.4)	3 (0.1)
Fragmented	25 (0.9)	25 (0.9)	190 (6.8)
Missing	29 (1.1)	28 (1.0)	160 (5.7)
Total	2799 (100)	2799 (100)	2799 (100)

Table S3. Analysis of single copy conserved genes using BUSCO v3.0.2 and the diptera gene set. Raw PacBio: primary contigs from the 3-cell *de novo* FALCON-Unzip assembly. Curated PacBio: Primary contigs after removal of bacterial contaminants and duplicated haplotypes. Previous reference from [18] GCA_000150765.1.

Table S4: Over 90% of genes formerly unassigned to chromosomes (e.g., residing on the UNKN chromosome) have now been placed to chromosomes and contigs. This table shows the contig that each UNKN gene is found in, as well as the chromosome that the gene is now assigned to.

gene	chrom	contig
AGAP012735	3R	000000F
AGAP012478	3R	000000F
AGAP012477	3R	000000F
AGAP012476	3R	000000F
AGAP012475	3R	000000F
AGAP012474	3R	000000F
AGAP012585	3R	000000F
AGAP012855	3R	000000F
AGAP012637	3R	000000F
AGAP012905	3R	000000F
AGAP012724	3R	000000F
AGAP012723	3R	000000F
AGAP012553	3R	000000F
AGAP012512	3R	000000F
AGAP012828	3R	000000F

AGAP012829	3R	000000F
AGAP012800	3R	000000F
AGAP012457	3R	000000F
AGAP012842	3R	000000F
AGAP012754	3R	000000F
AGAP012939	3R	000000F
AGAP012542	2R	000001F
AGAP012730	2R	000001F
AGAP012497	2R	000001F
AGAP012684	2R	000001F
AGAP012537	2R	000001F
AGAP012739	2R	000002F
AGAP012563	2R	000002F
AGAP012509	2R	000002F
AGAP012508	2R	000002F
AGAP013643	2R	000002F
AGAP012691	2R	000002F

AGAP012698	2R	000002F
AGAP012839	2R	000002F
AGAP012838	2R	000002F
AGAP012837	2R	000002F
AGAP012836	2R	000002F
AGAP012833	2R	000002F
AGAP012819	2R	000002F
AGAP012811	2R	000002F
AGAP012872	2R	000002F
AGAP028588	2R	000002F
AGAP012634	2R	000002F
AGAP012714	2R	000002F
AGAP012783	2R	000002F
AGAP012929	2R	000002F
AGAP012925	2R	000002F
AGAP012944	2R	000002F
AGAP012556	2R	000002F

AGAP012703	2R	000002F
AGAP012702	2R	000002F
AGAP012575	2R	000002F
AGAP012570	2R	000002F
AGAP012761	2R	000002F
AGAP012513	2R	000002F
AGAP012514	2R	000002F
AGAP012802	2R	000002F
AGAP012809	2R	000002F
AGAP012646	2R	000002F
AGAP012643	2R	000002F
AGAP012668	2R	000002F
AGAP012860	2R	000002F
AGAP012604	2R	000002F
AGAP012605	2R	000002F
AGAP012883	2R	000002F
AGAP012792	2R	000002F

AGAP012914	2R	000002F
AGAP012936	2R	000002F
AGAP012732	2R	000003F
AGAP012774	2R	000003F
AGAP012818	2R	000003F
AGAP012899	2R	000003F
AGAP012898	2R	000003F
AGAP012781	2R	000003F
AGAP012906	2R	000003F
AGAP012920	2R	000003F
AGAP012824	2R	000003F
AGAP012932	2R	000003F
AGAP012505	3L	000005F
AGAP012504	3L	000005F
AGAP012813	3L	000005F
AGAP012817	3L	000005F
AGAP012871	3L	000005F

AGAP028580	3L	000005F
AGAP028585	3L	000005F
AGAP012676	3L	000005F
AGAP012587	3L	000005F
AGAP012850	3L	000005F
AGAP012853	3L	000005F
AGAP012892	3L	000005F
AGAP012895	3L	000005F
AGAP012894	3L	000005F
AGAP012907	3L	000005F
AGAP012729	3L	000005F
AGAP012554	3L	000005F
AGAP012555	3L	000005F
AGAP012777	3L	000005F
AGAP012686	3L	000005F
AGAP012827	3L	000005F
AGAP012879	3L	000005F

AGAP012749	3L	000005F
AGAP012806	3L	000005F
AGAP012867	3L	000005F
AGAP012843	3L	000005F
AGAP012887	3L	000005F
AGAP012911	3L	000005F
AGAP012913	3L	000005F
AGAP012933	3L	000005F
AGAP012544	2R	000006F
AGAP012618	2R	000006F
AGAP012737	3R	000007F
AGAP012814	3R	000007F
AGAP012679	3R	000007F
AGAP012635	3R	000007F
AGAP012728	3R	000007F
AGAP012578	3R	000007F
AGAP012600	3R	000007F

AGAP012731	2R	000008F
AGAP012694	2R	000008F
AGAP012785	2R	000008F
AGAP012908	2R	000008F
AGAP012683	2R	000008F
AGAP012689	2R	000008F
AGAP012803	2R	000008F
AGAP012520	2R	000009F
AGAP012771	2L	000010F
AGAP012659	2L	000010F
AGAP012658	2L	000010F
AGAP028581	2L	000010F
AGAP012677	2L	000010F
AGAP012589	2L	000010F
AGAP012580	2L	000010F
AGAP012847	2L	000010F
AGAP012821	2L	000012F

AGAP012547	2R	000013F
AGAP012560	2R	000013F
AGAP012718	2R	000013F
AGAP012711	2R	000013F
AGAP012586	2R	000013F
AGAP012613	2R	000013F
AGAP012710	2R	000013F
AGAP012926	2R	000013F
AGAP012558	2R	000013F
AGAP012559	2R	000013F
AGAP012705	2R	000013F
AGAP012709	2R	000013F
AGAP012574	2R	000013F
AGAP012572	2R	000013F
AGAP012573	2R	000013F
AGAP012740	2R	000013F
AGAP012748	2R	000013F

AGAP012841	2R	000013F
AGAP012609	2R	000013F
AGAP012882	2R	000013F
AGAP012793	2R	000013F
AGAP012930	2R	000013F
AGAP012549	2R	000014F
AGAP012561	2R	000014F
AGAP012870	2R	000014F
AGAP012877	2R	000014F
AGAP012857	2R	000014F
AGAP012868	2R	000014F
AGAP012869	2R	000014F
AGAP012611	2R	000014F
AGAP012787	2R	000014F
AGAP012922	2R	000014F
AGAP012550	2R	000014F
AGAP012823	2R	000014F

AGAP012746	2R	000014F
AGAP012801	2R	000014F
AGAP012641	2R	000014F
AGAP012642	2R	000014F
AGAP012866	2R	000014F
AGAP012661	2R	000014F
AGAP012662	2R	000014F
AGAP012599	2R	000014F
AGAP012590	2R	000014F
AGAP012591	2R	000014F
AGAP012750	2R	000014F
AGAP012795	2R	000014F
AGAP012799	2R	000014F
AGAP012917	2R	000014F
AGAP012712	3R	000015F
AGAP012657	3R	000015F
AGAP012875	3R	000015F

AGAP012644	3R	000015F
AGAP012582	3R	000015F
AGAP012581	3R	000015F
AGAP012927	3R	000015F
AGAP012606	3R	000015F
AGAP012747	3R	000015F
AGAP012743	3R	000015F
AGAP012632	3R	000015F
AGAP012669	3R	000015F
AGAP012849	3R	000015F
AGAP012628	3R	000015F
AGAP012798	3R	000015F
AGAP012564	X	000016F
AGAP012675	X	000016F
AGAP012715	X	000016F
AGAP012511	X	000016F
AGAP012536	X	000016F

AGAP012535	X	000016F
AGAP012450	X	000016F
AGAP012888	X	000016F
AGAP012720	2R	000018F
AGAP012805	2R	000018F
AGAP012596	2R	000018F
AGAP012645	3R	000019F
AGAP012854	3L	000021F
AGAP012893	3L	000021F
AGAP012858	3L	000021F
AGAP012943	3L	000021F
AGAP012845	3L	000021F
AGAP012881	3L	000021F
AGAP028578	X	000022F
AGAP012545	3R	000023F
AGAP012796	3R	000023F
AGAP012758	2R	000025F

AGAP012812	2R	000025F
AGAP028586	2R	000025F
AGAP012859	2R	000025F
AGAP012856	2R	000025F
AGAP012713	2R	000025F
AGAP012901	2R	000025F
AGAP012928	2R	000025F
AGAP012726	2R	000025F
AGAP012571	2R	000025F
AGAP012769	2R	000025F
AGAP012820	2R	000025F
AGAP012598	2R	000025F
AGAP012620	2R	000025F
AGAP012938	2R	000025F
AGAP012501	3L	000027F
AGAP012773	3L	000027F
AGAP012489	3L	000027F

AGAP012863	3L	000027F
AGAP012593	3L	000027F
AGAP012456	3L	000028F
AGAP013599	2L	000029F
AGAP012695	2L	000029F
AGAP012699	2L	000029F
AGAP012529	2L	000029F
AGAP012528	2L	000029F
AGAP012775	2L	000029F
AGAP012685	2L	000029F
AGAP012530	2L	000029F
AGAP012531	2L	000029F
AGAP012804	2L	000029F
AGAP012660	2L	000029F
AGAP012759	2L	000029F
AGAP012934	2L	000029F
AGAP012756	3R	000030F

AGAP012690	3R	000030F
AGAP012666	3R	000030F
AGAP012434	3R	000030F
AGAP012433	3R	000030F
AGAP012437	3R	000030F
AGAP012436	3R	000030F
AGAP012435	3R	000030F
AGAP028651	3R	000030F
AGAP012603	3R	000030F
AGAP012822	3R	000030F
AGAP012636	3R	000030F
AGAP012633	3R	000030F
AGAP012755	3R	000030F
AGAP012655	3R	000031F
AGAP012673	3R	000031F
AGAP012765	3R	000031F
AGAP012921	3R	000031F

AGAP012649	3R	000031F
AGAP012623	3R	000031F
AGAP012915	3R	000031F
AGAP012664	3L	000032F
AGAP012770	3L	000032F
AGAP012865	3L	000032F
AGAP012640	3L	000033F
AGAP012638	3L	000033F
AGAP012579	3L	000033F
AGAP012565	3L	000034F
AGAP012566	3L	000034F
AGAP012473	3L	000034F
AGAP012472	3L	000034F
AGAP012492	3L	000034F
AGAP012471	3L	000034F
AGAP012470	3L	000034F
AGAP012810	3L	000034F

AGAP028583	3L	000034F
AGAP028584	3L	000034F
AGAP012469	3L	000034F
AGAP012671	3L	000034F
AGAP012670	3L	000034F
AGAP012861	3L	000034F
AGAP028704	3L	000034F
AGAP028705	3L	000034F
AGAP012780	3L	000034F
AGAP012688	3L	000034F
AGAP012626	3L	000034F
AGAP012886	3L	000034F
AGAP012797	3L	000034F
AGAP012656	2L	000035F
AGAP012525	2L	000035F
AGAP012527	2L	000035F
AGAP012523	2L	000035F

AGAP012496	2L	000035F
AGAP012734	2L	000035F
AGAP012612	2L	000035F
AGAP012462	2L	000035F
AGAP012763	2L	000035F
AGAP012524	2L	000035F
AGAP012707	2L	000035F
AGAP012708	2L	000035F
AGAP012495	2L	000035F
AGAP012490	2L	000035F
AGAP012493	2L	000035F
AGAP012487	2L	000035F
AGAP012534	2L	000035F
AGAP012825	2L	000035F
AGAP012826	2L	000035F
AGAP012465	2L	000035F
AGAP012498	2L	000035F

AGAP012486	2L	000035F
AGAP028807	2L	000036F
AGAP028810	2L	000036F
AGAP012725	2L	000037F
AGAP012653	3R	000038F
AGAP012830	3R	000038F
AGAP012674	3R	000038F
AGAP012654	3R	000038F
AGAP012706	3R	000038F
AGAP012680	3R	000038F
AGAP012681	3R	000038F
AGAP012682	3R	000038F
AGAP012807	3R	000038F
AGAP012764	3R	000038F
AGAP012736	2L	000040F
AGAP012831	2L	000040F
AGAP012873	2L	000040F

AGAP012569	2L	000040F
AGAP012896	2L	000040F
AGAP012903	2L	000040F
AGAP012924	2L	000040F
AGAP012667	2L	000040F
AGAP012937	2L	000040F
AGAP012562	3L	000041F
AGAP012692	3L	000041F
AGAP012716	3L	000041F
AGAP012678	3L	000041F
AGAP012576	3L	000041F
AGAP012577	3L	000041F
AGAP012900	3L	000041F
AGAP012594	3L	000041F
AGAP012627	3L	000041F
AGAP012885	3L	000041F
AGAP012697	3L	000042F

AGAP012696	3L	000042F
AGAP012557	3L	000042F
AGAP012851	3L	000042F
AGAP012852	3L	000042F
AGAP012789	3L	000042F
AGAP012503	3L	000042F
AGAP029093	3L	000042F
AGAP012808	3L	000042F
AGAP012648	3L	000042F
AGAP012631	3L	000042F
AGAP012844	3L	000042F
AGAP012647	3L	000042F
AGAP012880	3L	000042F
AGAP012910	3L	000042F
AGAP012639	3R	000045F
AGAP012786	3R	000045F
AGAP012687	3R	000045F

AGAP012693	3L	000047F
AGAP012584	3L	000047F
AGAP012788	3L	000047F
AGAP012567	2L	000049F
AGAP012651	2L	000049F
AGAP012650	2L	000049F
AGAP012616	2L	000049F
AGAP012902	2L	000049F
AGAP012941	2L	000049F
AGAP012617	2L	000049F
AGAP012779	2L	000049F
AGAP012568	2L	000049F
AGAP012546	3L	000050F
AGAP012652	3L	000050F
AGAP012834	3L	000050F
AGAP012832	3L	000050F
AGAP012704	3L	000050F

AGAP012816	3L	000050F
AGAP028589	3L	000050F
AGAP028587	3L	000050F
AGAP012719	3L	000050F
AGAP012772	3L	000050F
AGAP012742	3L	000050F
AGAP012753	3L	000050F
AGAP012602	3L	000050F
AGAP012741	3L	000050F
AGAP012918	3L	000050F
AGAP012919	3L	000050F
AGAP012931	3L	000050F
AGAP028799	3R	000051F
AGAP028829	X	000052F
AGAP028828	X	000052F
AGAP028986	X	000052F
AGAP028834	X	000052F

AGAP028990	X	000052F
AGAP028994	X	000052F
AGAP028859	X	000052F
AGAP028855	X	000052F
AGAP028856	X	000052F
AGAP028870	X	000052F
AGAP028837	X	000052F
AGAP028937	X	000052F
AGAP028936	X	000052F
AGAP028933	X	000052F
AGAP028919	X	000052F
AGAP028998	X	000052F
AGAP028956	X	000052F
AGAP028953	X	000052F
AGAP028952	X	000052F
AGAP028889	X	000052F
AGAP028885	X	000052F

AGAP028985	X	000052F
AGAP028951	X	000052F
AGAP028846	X	000052F
AGAP028864	X	000052F
AGAP028861	X	000052F
AGAP028863	X	000052F
AGAP028929	X	000052F
AGAP028909	X	000052F
AGAP012453	X	000052F
AGAP028962	X	000052F
AGAP029035	X	000052F
AGAP028895	X	000052F
AGAP028896	X	000052F
AGAP028793	X	000052F
AGAP028797	X	000052F
AGAP028794	X	000052F
AGAP028858	X	000052F

AGAP028912	X	000052F
AGAP028813	X	000052F
AGAP012722	3L	000057F
AGAP012835	3L	000057F
AGAP012619	3L	000057F
AGAP012768	3L	000057F
AGAP012608	3L	000057F
AGAP012607	3L	000057F
AGAP012767	3L	000057F
AGAP028423	3L	000057F
AGAP028826	2L	000058F
AGAP028897	2L	000058F
AGAP028839	2L	000058F
AGAP028833	2L	000058F
AGAP028993	2L	000058F
AGAP028853	2L	000058F
AGAP028883	2L	000058F

AGAP028948	2L	000058F
AGAP028800	2L	000058F
AGAP028821	2L	000058F
AGAP028820	2L	000058F
AGAP028823	2L	000058F
AGAP028822	2L	000058F
AGAP028825	2L	000058F
AGAP028849	2L	000058F
AGAP028848	2L	000058F
AGAP028867	2L	000058F
AGAP028862	2L	000058F
AGAP028868	2L	000058F
AGAP028924	2L	000058F
AGAP028927	2L	000058F
AGAP028922	2L	000058F
AGAP028903	2L	000058F
AGAP028900	2L	000058F

AGAP012451	2L	000058F
AGAP028963	2L	000058F
AGAP028943	2L	000058F
AGAP028947	2L	000058F
AGAP028944	2L	000058F
AGAP029032	2L	000058F
AGAP028892	2L	000058F
AGAP028811	2L	000058F
AGAP028792	2L	000058F
AGAP028790	2L	000058F
AGAP028928	2L	000058F
AGAP028814	2L	000058F
AGAP028815	2L	000058F
AGAP028816	2L	000058F
AGAP028817	2L	000058F
AGAP028818	2L	000058F
AGAP028819	2L	000058F

AGAP012543	2R	000059F
AGAP012541	2R	000059F
AGAP012439	2R	000059F
AGAP012518	2R	000059F
AGAP012540	2R	000059F
AGAP028996	2L	000060F
AGAP028991	2L	000060F
AGAP012428	2L	000060F
AGAP028803	2L	000060F
AGAP028842	2L	000060F
AGAP012432	2L	000060F
AGAP012430	2L	000060F
AGAP012429	2L	000060F
AGAP012431	2L	000060F
AGAP012521	3R	000062F
AGAP012522	3R	000062F
AGAP012464	3R	000062F

AGAP012454	2R	000063F
AGAP012447	2R	000063F
AGAP028577	2R	000063F
AGAP028576	2R	000063F
AGAP028806	2L	000066F
AGAP028805	2L	000066F
AGAP028804	2L	000066F
AGAP012458	3L	000071F
AGAP012904	3L	000071F
AGAP012721	2L	000073F
AGAP012945	2L	000073F
AGAP012595	2L	000073F
AGAP028995	2R	000074F
AGAP012552	2R	000074F
AGAP012622	2L	000081F
AGAP012459	2L	000081F
AGAP012757	2R	000083F

AGAP012745	2R	000083F
AGAP012665	2R	000083F
AGAP012717	2L	000085F
AGAP012519	2R	000087F
AGAP012438	2R	000088F
AGAP012488	2R	000088F
AGAP028987	2R	000088F
AGAP012442	2L	000089F
AGAP012441	2L	000089F
AGAP012760	2L	000089F
AGAP012440	2L	000089F
AGAP029002	2L	000093F
AGAP029003	2L	000093F
AGAP012782	2L	000093F
AGAP012784	2L	000093F
AGAP012625	2L	000093F
AGAP012515	2L	000093F

AGAP012516	2L	000093F
AGAP012517	2L	000093F
AGAP012624	2L	000093F
AGAP012876	3L	000095F
AGAP012663	3L	000095F
AGAP012891	2L	000096F
AGAP012449	2R	000104F
AGAP012484	2L	000105F
AGAP012538	2L	000105F
AGAP012506	2L	000105F
AGAP012481	2R	000106F
AGAP012610	2R	000106F
AGAP012912	2R	000106F
AGAP028997	X	000107F
AGAP028838	X	000107F
AGAP028835	X	000107F
AGAP028832	X	000107F

AGAP028830	X	000107F
AGAP028831	X	000107F
AGAP028992	X	000107F
AGAP028850	X	000107F
AGAP028851	X	000107F
AGAP028852	X	000107F
AGAP028854	X	000107F
AGAP028857	X	000107F
AGAP028872	X	000107F
AGAP028873	X	000107F
AGAP028876	X	000107F
AGAP028877	X	000107F
AGAP028874	X	000107F
AGAP028875	X	000107F
AGAP028878	X	000107F
AGAP028879	X	000107F
AGAP028836	X	000107F

AGAP028935	X	000107F
AGAP028934	X	000107F
AGAP028932	X	000107F
AGAP028930	X	000107F
AGAP028938	X	000107F
AGAP028911	X	000107F
AGAP028917	X	000107F
AGAP028954	X	000107F
AGAP028957	X	000107F
AGAP028882	X	000107F
AGAP028880	X	000107F
AGAP028984	X	000107F
AGAP028945	X	000107F
AGAP028921	X	000107F
AGAP028788	X	000107F
AGAP028789	X	000107F
AGAP028787	X	000107F

AGAP028802	X	000107F
AGAP028809	X	000107F
AGAP028808	X	000107F
AGAP028827	X	000107F
AGAP028959	X	000107F
AGAP028958	X	000107F
AGAP029034	X	000107F
AGAP029037	X	000107F
AGAP028843	X	000107F
AGAP028841	X	000107F
AGAP028840	X	000107F
AGAP028847	X	000107F
AGAP028845	X	000107F
AGAP028844	X	000107F
AGAP028988	X	000107F
AGAP028871	X	000107F
AGAP028866	X	000107F

AGAP028860	X	000107F
AGAP028869	X	000107F
AGAP028949	X	000107F
AGAP028925	X	000107F
AGAP028926	X	000107F
AGAP028920	X	000107F
AGAP028923	X	000107F
AGAP028906	X	000107F
AGAP028907	X	000107F
AGAP028902	X	000107F
AGAP028961	X	000107F
AGAP028965	X	000107F
AGAP028942	X	000107F
AGAP028940	X	000107F
AGAP028941	X	000107F
AGAP028946	X	000107F
AGAP028910	X	000107F

AGAP028916	X	000107F
AGAP028918	X	000107F
AGAP028898	X	000107F
AGAP028899	X	000107F
AGAP028894	X	000107F
AGAP028890	X	000107F
AGAP028891	X	000107F
AGAP028893	X	000107F
AGAP028798	X	000107F
AGAP028796	X	000107F
AGAP028950	X	000107F
AGAP028939	X	000107F
AGAP028884	X	000107F
AGAP028812	X	000107F
AGAP012444	3R	000111F
AGAP012445	3R	000111F
AGAP012443	3R	000111F

AGAP028575	3R	000111F
AGAP028579	3R	000119F
AGAP028931	2L	000120F
AGAP029033	2L	000120F
AGAP028989	2L	000120F
AGAP012776	2L	000124F
AGAP012766	3R	000128F
AGAP012762	2L	000133F
AGAP012588	3R	000142F
AGAP012494	2R	000145F
AGAP012532	2R	000145F
AGAP012533	2R	000154F
AGAP012500	2R	000155F
AGAP012499	2R	000155F
AGAP012583	2R	000206F
AGAP012526	2R	000206F
AGAP028915	UNKN	000243F

AGAP028914	UNKN	000243F
AGAP028824	X	000248F
AGAP028955	X	000248F
AGAP028887	X	000248F
AGAP028886	X	000248F
AGAP028881	X	000248F
AGAP012700	2L	000283F
AGAP012752	2L	000283F
AGAP012548	not placed	N/A
AGAP012738	not placed	N/A
AGAP012727	not placed	N/A
AGAP028913	not placed	N/A
AGAP012507	not placed	N/A
AGAP029100	not placed	N/A
AGAP012751	not placed	N/A
AGAP012874	not placed	N/A
AGAP012778	not placed	N/A

AGAP012815	not placed	N/A
AGAP028582	not placed	N/A
AGAP012468	not placed	N/A
AGAP012467	not placed	N/A
AGAP012461	not placed	N/A
AGAP012672	not placed	N/A
AGAP012460	not placed	N/A
AGAP028268	not placed	N/A
AGAP012448	not placed	N/A
AGAP012897	not placed	N/A
AGAP028295	not placed	N/A
AGAP028888	not placed	N/A
AGAP012466	not placed	N/A
AGAP028786	not placed	N/A
AGAP012455	not placed	N/A
AGAP012551	not placed	N/A
AGAP012446	not placed	N/A

AGAP012909	not placed	N/A
AGAP028801	not placed	N/A
AGAP012923	not placed	N/A
AGAP012502	not placed	N/A
AGAP012940	not placed	N/A
AGAP012615	not placed	N/A
AGAP012701	not placed	N/A
AGAP028865	not placed	N/A
AGAP028650	not placed	N/A
AGAP013132	not placed	N/A
AGAP012485	not placed	N/A
AGAP012539	not placed	N/A
AGAP012744	not placed	N/A
AGAP012483	not placed	N/A
AGAP028908	not placed	N/A
AGAP028904	not placed	N/A
AGAP028905	not placed	N/A

AGAP028901	not placed	N/A
AGAP012733	not placed	N/A
AGAP012862	not placed	N/A
AGAP028574	not placed	N/A
AGAP012452	not placed	N/A
AGAP028960	not placed	N/A
AGAP028964	not placed	N/A
AGAP012592	not placed	N/A
AGAP012597	not placed	N/A
AGAP012848	not placed	N/A
AGAP012840	not placed	N/A
AGAP012846	not placed	N/A
AGAP012601	not placed	N/A
AGAP012621	not placed	N/A
AGAP029036	not placed	N/A
AGAP029038	not placed	N/A
AGAP012889	not placed	N/A

AGAP028246	not placed	N/A
AGAP028791	not placed	N/A
AGAP028795	not placed	N/A
AGAP012884	not placed	N/A
AGAP012463	not placed	N/A
AGAP012794	not placed	N/A
AGAP012916	not placed	N/A
AGAP012935	not placed	N/A

avro==1.8.2
certifi==2018.4.16
chardet==3.0.4
ConsensusCore==1.0.2
ConsensusCore2==3.0.0
Cython==0.28.5
decorator==4.3.0
edlib==1.2.3.post1
falcon-kit==1.2.2+git.00e8272b663d32a0962ae92ab92324a3b3eb4b46
FALCON-pbsmrtpipe==1.0.0
FALCON-polish==1.0.0
falcon-unzip==1.1.2+git.354e53c5beb103c5c8301914d86fe96364b29288
functools32==3.2.3.post2
future==0.16.0
GenomicConsensus==2.2.2
idna==2.7
intervaltree==2.1.0
iso8601==0.1.12
jsonschema==2.6.0
msgpack==0.5.6
msgpack-python==0.5.6
networkx==2.1
numpy==1.15.0
pbalign==0.3.0
pbcommand==1.1.2
pbcore==1.5.4
pbcoretools==0.2.4
pypeflow==2.0.4+git.005acb16689c18c09cf552b42911e69629ffeceb
pysam==0.13
pytz==2018.5
requests==2.19.1
sortedcontainers==2.0.4
urllib3==1.23
xmlbuilder==1.0

Note S1.
Software versions for *de novo* assembly with FALCON-Unzip.

```
[job.defaults]
njobs = 100
submit = qsub -S /bin/bash -sync y -V  \
  -q ${JOB_QUEUE}     \
  -N ${JOB_NAME}        \
  -o "${JOB_STDOUT}" \
  -e "${JOB_STDERR}" \
  -pe smp ${NPROC}    \
  "${JOB_SCRIPT}"

JOB_QUEUE = default7
MB = 30000
NPROC = 6
[General]
input_type = raw
input_fofn = fasta.fofn
pwatcher_type=blocking

genome_size = 280000000
seed_coverage = 40
length_cutoff = -1
length_cutoff_pr = 5000
falcon_greedy = False
falcon_sense_greedy=False

pa_daligner_option = -e0.76 -l1200 -k18 -h70 -w8 -s100
ovlp_daligner_option = -k24 -h1024 -e.95 -l1800 -s100
pa_HPCdaligner_option = -v -B128 -M24
ovlp_HPCdaligner_option = -v -B128 -M24
pa_HPCTANmask_option = -k18 -h480 -w8 -e.8 -s100
pa_HPCREPmask_option = -k18 -h480 -w8 -e.8 -s100
pa_DBsplit_option = -x500 -s400
ovlp_DBsplit_option = -s400

falcon_sense_option = --output-multi --min-idt 0.70 --min-cov 3 --max-n-read 400 --n-core 24
overlap_filtering_setting = --max-diff 100 --max-cov 150 --min-cov 3 --n-core 24

[job.step.da]
NPROC=4
MB=20000
njobs=100
[job.step.la]
NPROC=8
MB=40000
njobs=100
[job.step.cns]
NPROC=8
MB=40000
njobs=100
```

```
[job.step.pda]
NPROC=8
MB=40000
njobs=100
[job.step.pla]
NPROC=4
MB=20000
njobs=100
[job.step.asm]
NPROC=24
MB=120000
```

Note S2.
Configuration file for FALCON assembly.

```
[General]

[Unzip]
input_fofn= fasta.fofn
input_bam_fofn=bam.fofn

[job.defaults]
pwatcher_type=blocking
max_n_open_files = 1000
njobs=50
JOB_QUEUE=bigmem
NPROC=4
MB=32000
submit = qsub -S /bin/bash -sync y -V  \
  -q ${JOB_QUEUE}     \
  -N ${JOB_NAME}        \
  -o "${JOB_STDOUT}" \
  -e "${JOB_STDERR}" \
  -pe smp ${NPROC}    \
  "${JOB_SCRIPT}"
[job.step.unzip.track_reads]
njobs=1
NPROC=24
MB=192000
[job.step.unzip.blasr_aln]
njobs=45
NPROC=24
MB=192000
[job.step.unzip.phasing]
njobs=100
NPROC=2
MB=16000
[job.step.unzip.hasm]
njobs=1
NPROC=48
MB=384000
[job.step.unzip.quiver]
njobs=45
NPROC=24
MB=192000
```

Note S3.
Configuration file for FALCON-Unzip.
